# Ultrasound and Microwave-Assisted Synthesis of Hexagonally Ordered Ce-Promoted Mesoporous Silica as Ni Supports for Ethanol Steam Reforming

**DOI:** 10.3390/nano13060997

**Published:** 2023-03-09

**Authors:** Jorge Tovar-Rodriguez, Emiliano Fratini, Piero Baglioni, Carlo Ferrari, José Antonio de los Reyes-Heredia, Yonatan Ramírez-Hernández, Ignacio René Galindo-Esquivel

**Affiliations:** 1 Department of Chemistry “Ugo Schiff” and Center for Colloid and Surface Science (CSGI), University of Florence, Via della Lastruccia 3, 50019 Florence, Italy; 2 National Institute of Optics (INO–UOS Pisa), National Council of Research (CNR), Via Giuseppe Moruzzi 1, 56124 Pisa, Italy; 3 Process Engineering and Hydraulics Department, Metropolitan Autonomous University, UAM, Av. San Rafael Atlixco 186, Ciudad de México 09340, Mexico; 4 Chemical Engineering Department, University of Guanajuato, Noria Alta S/N, Noria Alta, Guanajuato 36050, Mexico

**Keywords:** Cerium catalyst, green chemistry, MCM-41, mesoporous materials, microwave chemistry, nickel catalyst, SAXS

## Abstract

Solvothermal synthesis of mesoporous materials based on amphiphilic molecules as structure-directing agents can be enhanced using non-conventional technologies for stirring and thermal activation. Here, we disclose a green synthesis approach for the preparation of cerium-modified hexagonally ordered silica sieves. Ultrasound micromixing enabled us to obtain well-dispersed Ce in the self-assembled silica network and yielded ordered materials with high cerium content (Ce/Si molar ratio = 0.08). Microwave dielectric heating, applied by an innovative open-end coaxial antenna, was used to reduce the overall hydrothermal synthesis time and to improve the surface area and textural properties. These mesoporous materials were used as a Ni catalyst support (10 wt.% metal loading) for the ethanol steam reforming reaction. The new catalysts featured complete ethanol conversion, high H_2_ selectivity (65%) and better stability, compared to the same catalyst prepared with magnetic stirring and conventional heating. The Ce-promoted silica sieves offered a suitable support for the controlled growth of nanocarbon that does not result in catalyst deactivation or poisoning after 6 h on stream.

## 1. Introduction

Ordered mesoporous sieves are a versatile class of template-based materials that can find multiple applications as catalytic supports [1], as adsorbing matrixes [2,3], as enzyme substrates for immobilization [4] and as photocatalytic systems holders [5]. In particular, silica-based MCM-41 (Mobil Composition of Matter No. 41) exhibits hexagonal ordering, high surface area and narrow pore size distribution [6], but it is seldom used as such since it lacks any catalytic activity. Several examples can be found in the literature concerning the structural modification of mesoporous silica, either by direct or post-synthesis functionalization, to provide catalytic activity. Silica sieves have been used for oxidative, acid-base, and photo catalysis or as supports for a metal active site [7,8,9]. Therefore, it is important to develop green non-conventional synthesis routes [10] that make possible structure modification and tailoring its characteristics in a minimum number of steps, while preserving the features of the parent material.

In this regard, ultrasound (US) represents an effective and low-cost alternative to magnetic and mechanical stirring, shaking and mixing [11]. US-induced acoustic cavitation has been demonstrated to improve the nucleation and crystallization steps during the solvothermal synthesis of template-based micro and mesoporous materials such as zeolites or zeotypes [12]. US can accelerate chemical reactions, modify the viscosity and diffusion of the reaction media and permit the use of less extreme conditions, which makes it a safe and green technology. Still, the sonochemical synthesis of metal-modified mesoporous silica is scarcely reported. On the other hand, microwaves (MW) assisted chemistry is a useful tool for thermal activation to reduce the long synthesis time, which is typically required to produce ordered nanoporous materials by hydrothermal treatment [13]. Depending on the dielectric properties of the reaction media and solvents, it is possible to achieve shorter synthesis times by a more effective heating and temperature profile, produced by MW irradiation. This is particularly attractive for the design of green synthesis approaches since it is possible to reduce the energetic inputs, the number of involved steps and the overall cost. Nonetheless, most of the commercially available reactors for MW-synthesis have a traditional closed-box configuration, which makes them particularly unsuitable for scaling-up and may limit their operative conditions or coupling with other techniques [14].

Given its oxygen vacancies and oxygen storage capacity [15], cerium oxide and cerium-based composite materials [16] are a well-known and preferred catalyst for oxidation of reagents. For instance, cerium modified mesoporous silicas have been successfully employed for the oxidation of organic compounds [17], exhibiting negligible pore diffusion resistance, higher selectivity, improved catalytic activity and good catalyst reusability [18], compared to Ce over amorphous SiO_2_, CeO_2_ or other cerium-based supports. Furthermore, Ce-promoted hexagonally ordered materials have also been used as catalytic supports for metallic active phases in the reforming reactions of hydrocarbons to produce hydrogen, since their oxidation capacity makes them less prone to carbon deposition, carbon poisoning and consequent catalyst deactivation [19,20,21].

The growing interest in hydrogen as an alternative energy carrier is based on its efficiency and near zero-emissions nature, particularly when obtained from reforming of renewable hydrocarbons such as bioethanol. Several noble metals, such as Ru, Pd, Ag, Pt and Rh [9,22,23], have been demonstrated to be adequate catalysts for the ethanol steam reforming reaction. However, their widespread use still faces the high costs and limited availability associated with materials based on noble metals. In order to address this issue, transition metal catalysts such as Ni and Cu represent a cheap and effective alternative [24]. Nevertheless, Ni catalysts tend to deactivate swiftly, as they seem to promote ethylene, acetaldehyde and acetone formation, all of which have been identified as coke precursors [25]. For this reason, it is necessary to design catalysts that enhance the synergy between the active phase and the support to improve hydrogen yields and inhibit carbon deposition.

Herein, we report the isomorphic incorporation of cerium into hexagonally ordered silica by applying US during the colloidal growth of MCM-41 in a direct approach. Once optimal conditions were found for improved hexagonal ordering, MW irradiation was applied for thermal activation by means of an innovative coaxial applicator to reduce the hydrothermal synthesis time. To the best of our knowledge, no other reports can be found regarding the US-MW Ce-modification of mesoporous silica. Cerium distribution and location in the hexagonal structure was assessed by several characterization techniques. The effect of structural and chemical change on their catalytic performance was evaluated by using them as supports for a nickel catalyst, in the hydrogen production through ethanol steam reforming (ESR).

## 2. Materials and Methods

### 2.1. Catalyst Synthesis

Cerium isomorphic substitution in mesoporous silica was achieved in a direct synthesis approach. The structure-directing agent was the cationic amphiphilic molecule hexadecyltrimethylammonium bromide (CTABr, CH_3_(CH_2_)_15_N(Br)(CH_3_)_3_ Sigma-Aldrich, St. Louis, MO, USA). Ammonium hydroxide was used as mineralizing agent (30% NH_3_ wt/v Sigma); cerium nitrate was used as Ce precursor (Ce(NO_3_)_3_•6(H_2_O), Sigma-Aldrich, St. Louis, MO, USA) and tetraethyl orthosilicate (TEOS, (CH_3_CH_2_O)_4_Si, Sigma-Aldrich, St. Louis, MO, USA) was used as silicon source. All reagents were used without further purification. A series of materials with the following molar composition of the synthesis colloid were prepared:

1.0. (CH_3_CH_2_O)_4_Si: x Ce(NO_3_)_3_•6H_2_O: 0.3CTABr: 18NH_3_: 160H_2_O; x = 0.02, 0.04, 0.06 and 0.08.

The materials were named xCe-MCM-41, where x indicates the Ce/Si nominal molar ratio. US was used for micromixing during the nucleation step to increase Ce dispersion in the mesoporous structure, and MW irradiation was applied for thermal activation. MW-assisted hydrothermal synthesis was carried out in a stainless-steel high-pressure reactor using a coaxial antenna for in-situ MW application. Details concerning the design, construction of the reactor [26] and the technical specifications for the MW applicator are reported elsewhere [14]. Conventional hydrothermal synthesis was performed in a Teflon-lined stainless-steel reactor using a heating mantle.

In a typical batch, CTABr (2.85 g) was dissolved in deionized water (50 mL) and NH_4_OH (33.5 mL). This solution was kept at constant temperature (318 K) and vigorous stirring (500 rpm) until complete dissolution of the surfactant was achieved. A second solution, containing the required amount of cerium nitrate dissolved in deionized water (33.5 mL), was mixed with the surfactant solution. Then, the silicon precursor (4.56 mL TEOS) was added dropwise to the mixture. This colloid was treated with US for another 2.5 h (Frequency 40 kHz), before being aged for an additional 4 h. The samples were treated hydrothermally at 373 K and autogenous pressure for 24 h (conventional synthesis) and 1 h (for MW-assisted synthesis). The precipitated product was separated by centrifugation, washed with deionized water, and dried at 353 K overnight. The obtained powder was annealed at 823 K and atmospheric pressure under static air for 4 h to remove the surfactant and to yield a porous material.

Ni catalysts were prepared by wet impregnation method to achieve a total loading of 10 wt% of Ni over the catalyst, using a solution of Ni(NO_3_)_2_•6H2O (Aldrich). The resulting solids were dried at room temperature for 24 h and then calcined at 823 K for 2 h. Hereafter, the catalysts containing 10 wt% of Ni are referred to as Ni/xCe-MCM-41, using the values for x depending on the nominal amount of Ce molar load in the support.

### 2.2. Catalyst Characterization

SAXS measurements were carried out with a HECUS S3-MICRO camera (Kratky-type) equipped with a position-sensitive detector (OED 50M) containing 1024 channels of width 54 μm. Cu Kα radiation of wavelength λ = 1.542 Å was provided by an ultra-brilliant point micro-focus X-ray source (GENIX-Fox 3D, Xenocs, Grenoble), operating at a maximum power of 50 W (50 kV and 1 mA). The sample-to-detector distance was 281 mm. The volume between the sample and the detector was kept under vacuum during the measurements to minimize scattering from the air. The Kratky camera was calibrated in the small angle region using silver behenate (d = 58.34 Å) [27]. Scattering curves were obtained in the *Q*-range between 0.05 and 0.5 Å**^−^**^1^, assuming that *Q* is the scattering vector, *Q = 4π/λ × sinθ*, and *2θ* is the scattering angle. Calcined powder samples were placed into a 1 mm demountable cell having Kapton foils as windows. For in-situ measurements during colloidal ageing, liquid samples were mounted in a capillary tube (80 mm length, 1.0 mm diameter, 0.1 mm wall thickness). The temperature was set at 298 K for solid samples and 318 K for colloidal sample measurements, and it was controlled by a Peltier element with an accuracy of 0.1 K. All scattering curves were corrected for the empty cell or capillary contribution considering the relative transmission factor. X-ray diffraction measurements were performed in a Bruker-D8 Advance X-ray powder diffractometer in the Bragg–Brentano *θ-2θ* geometry. The X-ray source was Cu Kα radiation, operating at 40 kV and 30 mA with a wavelength of λ = 1.54 Å. The detection was carried out using a Lynx-eye linear-type detector, in the 2θ interval from 1 to 70 degrees. Attenuated Total Reflectance Fourier Transform infrared spectra were obtained for samples before and after being calcined, in the wavenumber interval from 600 to 4000 cm^−1^ with an Infrared Spectrometer Thermo Nicolet Nexus 4700 FT-IR ATR. UV-vis spectra of the Ce modified samples were acquired by using a Spectrometer (Perkin Elmer USA Lambda 3S) in the wavelength interval of 200 to 600 nm, using MgO as reference. The ^29^Si Solid-state Nuclear Magnetic Resonance spectra (MAS-NMR) of the powder samples were recorded using a Bruker AVANCE II NMR Spectrometer with a CPMAS H-X BB detector of 4 mm, operating at a resonance frequency of 59 MHz. The spin velocity of the sample was set at 5 kHz and the recycling time between experiments was 10 s using the High-Power Decoupling, Cross-Polarization technique (HP-DEC). High resolution Transmission Electron Microscopy (HR-TEM) was performed with a JEOL 2100F equipment, using a lighting source of 200 kV of acceleration and a high vacuum system, to obtain both TEM and High Angle Annular Dark Field (HAADF) micrographs. Nitrogen adsorption and desorption isotherms were acquired using a Beckman Coulter SA-3100 Surface area analyzer. Calcined samples were outgassed prior to analysis in vacuum conditions at a temperature of 473 K until a pressure of 1.3 Pa was reached. Morphology and particle size of the catalysts were analyzed using a SIGMA Field Emission Scanning Microscope (Carl Zeiss Microscopy GmbH, Germany). The micrographs were acquired from uncoated samples, using the In-Lens Secondary Electron detector and the Backscattered Electron Diffraction (EBDS) simultaneously to yield Z-contrast micrographs of the catalysts.

### 2.3. Catalytic Evaluation

The ethanol steam reforming reaction was performed in a tubular continuous reactor (10 mm internal diameter, 50 cm length). A catalyst sample (50 mg) was placed in the reactor in a powder quartz bed. A thermocouple K-type was placed near the catalytic bed to control the reaction temperature. The catalysts were reduced in-situ at 823 K for 2 h with a hydrogen flow (50 cm^3^ min**^−^**^1^), using a heating rate of 10 K min**^−^**^1^. After reduction, the system was flushed with argon (50 cm^3^ min**^−^**^1^) for 15 min. The reforming reactions were performed at 723 and 773 K under atmospheric pressure. A liquid flow containing water and ethanol in a molar ratio of 3:1 was evaporated in argon and was fed at a rate of 0.01 cm^3^ min**^−^**^1^. The ethanol and water streams were mixed and adjusted with pure argon until a total flow of 100 cm^3^ min**^−^**^1^ was measured. The composition of the reactor effluent was analyzed by two gas chromatographs Clarus 580 (Perkin Elmer); one equipped with a Q-plot capillary column and an FID detector was used for the separation of CH_4_, C_2_H_5_OH, CH_3_CHO, C_2_H_4_, CH_3_COOH, and CH_3_COCH_3_. The other gas chromatograph was equipped with a Molsieve capillary column and a TCD detector for the analysis of H_2_, CO, CH_4_, CO_2_ and C_2_H_4_. The two detectors were calibrated to extract the molar fraction of the different gases.

## 3. Results and Discussion

### 3.1. Effect of Ultrasound and Microwave Irradiation on the Characteristics of Ce-Promoted Mesoporous Silica

**Figure 1:**
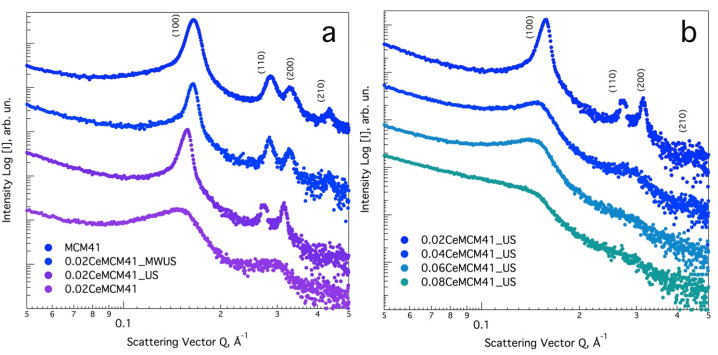
Small Angle X-ray Scattering curves for (**a**) MCM-41 and Ce-modified MCM-41 (Ce/Si molar ratio = 0.02) prepared using US, MW irradiation and conventional magnetic stirring (0.02Ce-MCM-41); (**b**) SAXS curves for Ce-modified silica with ranging Ce/Si molar ratios.

Small Angle X-ray Scattering (SAXS) curves were recorded in the low Q scattering vector interval from 0.05 to 0.5 Å^−1^, to provide information concerning the structure and hexagonal ordering of the cerium modified silicas. Figure 1a,b show the SAXS patterns for all the xCe-MCM-41 powder samples after calcination. Pure siliceous mesoporous MCM-41 exhibits four well-defined diffraction peaks, centered at *Q* vector values of 0.165, 0.285, 0.329 and 0.435 Å^−1^. These scattered signals can be associated to the first (*hlk*) planes, where *hlk* is equal to (100), (110), (200) and (210), indexed to the hexagonal system, space group *p6mm* [28]. Table 1 summarizes the interplanar distance d_100_, calculated using the corresponding *Q* vector value and Bragg′s law, *λ = 2d_hlk_ × sinθ*.

After cerium loading, a shift towards lower reciprocal *Q* values is observed for all samples, which is indicative of larger d_100_ spacing values, as consequence of Ce direct incorporation (Table 1, d_100_ entry). In a previous publication [29], we demonstrated that the broadening of the d_100_ interplanar distance depends on the type of substituted heteroatom and the amount effectively incorporated in the silica framework. For the lowest Ce/Si molar ratio, three well-resolved Bragg diffraction peaks are featured (Figure 1b, 0.02Ce-MCM-41_US), whereas a higher cerium content results in broader scattering curves. Therefore, the d_100_ interplanar spacing enlarges from 4.42 nm to a maximum value of 5.04 nm (for Ce/Si = 0.08).

This evolution towards less ordered sieves could be caused by the disparity of covalent radii between Si (1.11 Å) and Ce (2.04 Å) [30]. If Ce incorporation into the silicon structure occurs in isomorphic manner, as the Ce/Si molar ratio increases, the substitution of Si-O-Si for larger Si-O-Ce bonding results in materials with less-resolved diffraction patterns. This covalent radius disproportion also accounts for larger lattice cell a0 values (Table 1, a_0_ entry). For the hexagonal structure, the cell constant can be estimated as a_0_ = 2d_100_/√3 and its value ranges from 5.10 to 5.82 nm (for 0.08Ce-MCM-41_US).

A comparative study was performed to assess the effect of US and MW on the hexagonal regularity of samples with a fixed Ce/Si ratio of 0.02 (Figure 1a). It is noteworthy that a material prepared using magnetic stirring exhibited a broad d_100_ peak centered on *Q* = 0.152 Å^−1^; then, a secondary less intense peak, which could be interpreted as the convolution of the d_110_ and d_200_ reflections, is centered at *Q* = 0.297 Å^−1^. In contrast, a sample prepared using US during colloidal ageing (0.02Ce-MCM_US) featured three Bragg peaks in the explored low *Q* interval (d_100_ = 4.63 nm). This suggests that the micromixing produced by acoustic cavitation effectively promoted better Ce dispersion and incorporation in the silica network during nucleation, compared to conventional magnetic stirring. Once the colloids were aged, solvothermal synthesis was enhanced by an innovative open-end coaxial dipole applicator [14] for in-situ MW irradiation in a high-pressure stainless-steel reactor [26]. Figure S1 (Electronic Supplementary Information) shows a typical temperature profile for the hydrothermal preparation of mesophases using MW for thermal activation (0.02Ce-MCM-41_MWUS sample). Under this practical approach, 373 K can be achieved within two minutes of applied power (operating at a maximum power of 240 W); then, a PID controller keeps a variable supply of around 60 W to maintain the desired operating temperature. Similarly, a well-defined SAXS diffraction curve was recorded for the material prepared using MW irradiation. The interplanar distance calculated for this sample was the closest to the MCM-41 parent material (4.46 nm).

Further, the coaxial antenna is a green and effective alternative, compared to the typically used closed-box MW ovens and digestors, as it consumes less than 10 % the power required in the latter devices to achieve and sustain the same temperature. This homogeneous heating source not only allowed a smooth control of the reaction parameters, but also yielded a material with a high degree of ordering after calcination while reducing the required time for conventional heating by one order of magnitude (1 h compared to 24 h).

**Figure 2:**
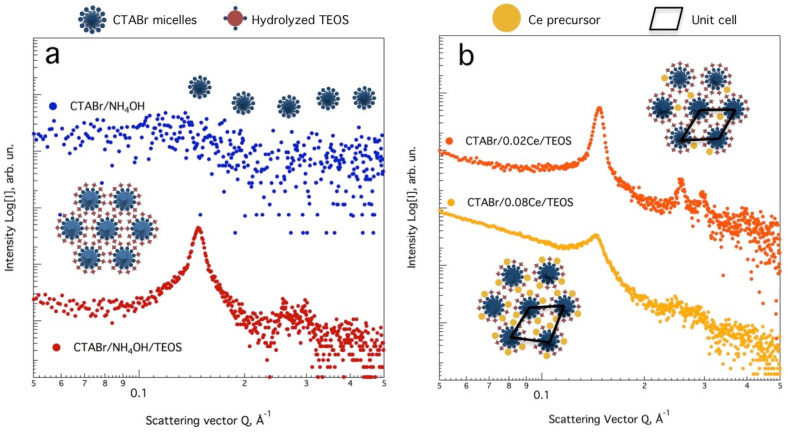
In-situ SAXS measurements for (**a**) CTABr solution, MCM-41 synthesis colloid, and (**b**) colloidal Ce-modified mesoporous silica (using 0.02 and 0.08 Ce/Si molar ratios). Inset graphs, possible formation mechanism for the isomorphic incorporation of the Ce species.

To better understand the formation mechanism of the cerium-promoted hexagonal silica framework, SAXS curves for the synthesis colloid were measured within a capillary cell in the liquid state, along the same *Q* interval. Figure 2a shows the SAXS curves for the surfactant solution and the MCM-41 synthesis colloid. Under the acquisition parameters (50 W, t_acq_ = 1800 s), the amphiphilic cationic surfactant solution in NH_4_OH does not produce any scattering pattern typical of an ordered structure (upper curve). Upon alkoxide addition, given the strong alkaline conditions (pH = 12.7), the tetraethyl orthosilicate molecules are rapidly hydrolyzed and interact with the positively charged cationic micelles. For the CTAB/TEOS system (lower curve), a main Bragg peak can be observed with secondary less-defined peaks, suggesting that the hexagonal arrangement of the micelles occurs immediately after TEOS hydrolysis and silica oligomer formation (TEOS/CTAB molar ratio 1:0.30). Figure 2b shows the SAXS curves for colloidal Ce-modified silica using 0.02 and 0.08 Ce/Si molar ratios. For the lowest Ce content, the main Bragg peak is located at 4.91 Å^−1^, whereas peak position is shifted to 5.05 Å^−1^ for the colloidal system with the highest Ce/Si molar ratio. After calcination, the peaks maxima change to even lower reciprocal values because of thermal shrinkage [31] and the intensity of all Bragg reflections is reduced once the template is removed. Inset Figure 2b shows the lattice cell and a possible formation mechanism for Ce-modified hexagonal sieves. For the lowest Ce/Si molar ratio, the position and intensity of the (100) reflection and the presence of secondary Bragg peaks suggest a highly ordered material (upper curve). Therefore, US-enhanced mixing was helpful in achieving Ce distribution during silica self-assembly. On the other hand, at higher Ce/Si ratios the amount of cerium precursor and a larger concentration of counter ions (NO_3_^−^) could be responsible for hindering hexagonal ordering by modifying micelle aggregation and density numbers [32]. The amount of Ce effectively incorporated and the difference between covalent radii could be responsible for the unit cell deformation. A poor Ce distribution along the silica sieve results in a non-homogeneous Ce incorporation, which produces an uneven stretching of the primitive cell, and therefore modifies its shape and geometry.

In a previous report [33], optimal values for surfactant/Si and Ce/Si molar ratios were found for Ce substituted MCM-41 (0.2 and 0.025, respectively), by analyzing the X-ray scattering and relative intensity of the d_100_ plane. A Ce-MCM-41 sample prepared under magnetic stirring with a Ce/Si molar ratio of 0.025, exhibited only the scattering of the first two *hlk* planes. Considering the SAXS curves for 0.02Ce/Si sample, the US-assisted methodology here reported resulted in a material with better cerium dispersion along the hexagonal framework and a higher degree of ordering. The latter also holds true for materials prepared under non-hydrothermal methodologies [34], and materials prepared using oven-type MW-assisted synthesis [35].

**Figure 3:**
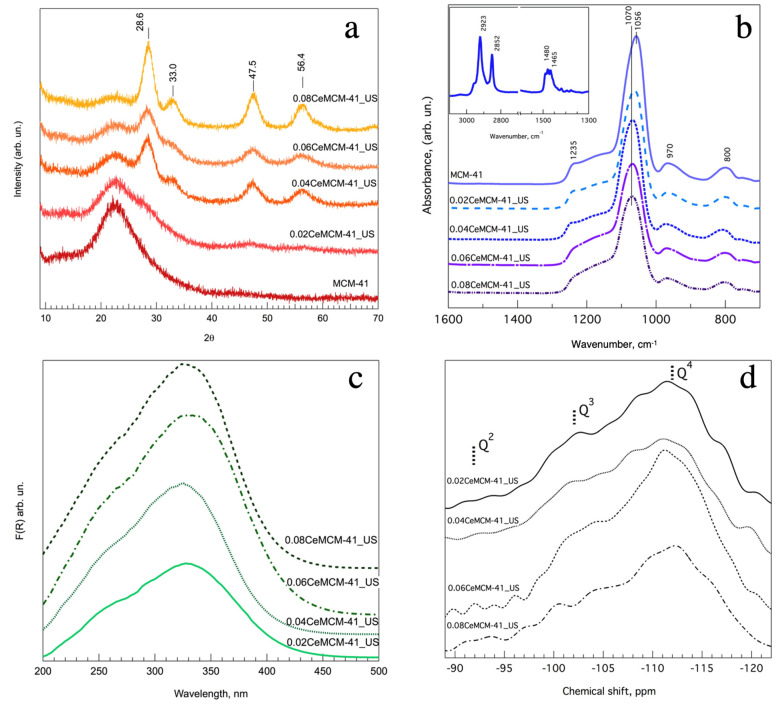
(**a**) X-ray diffraction curves for xCe-MCM-41_US materials. (**b**) FT-IR ATR spectra for calcined cerium-modified mesoporous silica (inset: as-prepared MCM-41). (**c**) UV-visible Diffuse Reflectance spectra for Ce-promoted silica sieves. (**d**) ^29^Si solid-state HPDEC MAS-NMR spectra of cerium modified mesoporous silica xCe-MCM-41.

To further characterize the Ce content in all samples, XRD curves for the xCe-MCM-41_US materials were recorded in the *2θ* interval from 10 to 70° (Figure 3a). Unmodified mesoporous silica and the samples with lowest Ce/Si molar ratio exhibit a single broad peak, encompassing *2θ* values from 18 to 26°. For Ce/Si values of 0.04 and higher, four diffraction peaks are observed at around *2θ* values of 28.6, 33, 47.5 and 56.4°, indexed to the Bragg scattering of the (111), (200), (220) and (311) planes for the cubic phase in CeO_2_. Sharper and more intense diffraction peaks were observed for the sample with the highest Ce content (0.08Ce-MCM-41_US). Therefore, when the Ce/Si molar ratio exceeds 0.02 CeO_2_ crystals are formed. In conjunction with the SAXS results, these findings lead to some conclusions concerning the possible mechanism of Ce incorporation into the hexagonally ordered mesoporous silica. For low metal loadings, the well-preserved hexagonal structure is indicative of a successful Ce dispersion and isomorphic incorporation in the SiO_2_ structure, promoted using US acoustic cavitation and MW irradiation. Higher Ce/Si molar ratios could favor the deposition of cubic CeO_2_ particles outside the silica framework as a secondary incorporation mechanism.

The FT-IR ATR spectra for all xCe-MCM-41_US materials are shown in Figure 3b. Silica MCM-41 features three main bands that can be assigned to the asymmetrical stretching vibration (ν_as_) of the Si-O-Si bond, located at wavenumber values of 1235, 1056 and 970 cm^−1^. For all cerium modified materials, the main band located at 1056 shifts to 1070 cm^−1^ while the intensity of the band located at 1230 cm^−1^ is reduced as the molar ratio Ce/Si increases. This could also suggest a lower recurrence of Si-O-Si that could be replaced by larger Si-O-Ce bonds. Concerning the quaternary amine molecule, inset Figure 3b features two signals at about 1465 and 1480 cm^−1^, which can be assigned to the δ(CH_2_) and δ_as_(N-CH_3_) bending modes of the surfactant molecule within the pores (scissoring region), whereas bands at 2852 and 2923 cm^−1^ belong to the symmetric and asymmetric (ν_sym_(CH_2_) and ν_as_(CH_2_)) stretching modes of the surfactant alkyl chain [36]. After calcination these bands are no longer present in the spectrum, as calcination removed the template effectively. The location and intensity of the band centered at 800 cm^−1^, attributed to the vibrations of the ν_s_(Si-O-Si) does not change because of cerium incorporation.

In order to determine the coordination of the Ce species in the hexagonal silica framework, the UV-visible Diffuse Reflectance Spectra for all calcined samples was recorded. Figure 3c shows a single broad band in the wavelength interval from 200 to 400 nm for all samples with a maximum value at around 330 nm. The intensity of this band increases with the amount of Ce introduced in the sample. For crystalline CeO_2_, there is a band gap of 3.1 eV (3.7 eV at 330 nm for these materials). Since electronic transitions in the hexa-coordinated atom (Ce^3+^) require more energy than the transitions for the tetra-coordinated atom (Ce^4+^), it is possible to assign this band to the transitions Ce-O for Ce^4+^ [37]. Regardless of the position and distribution of Ce in the hexagonal silicon structure, the nature of the cerium species is tetra-coordinated for all cases.

The ^29^Si NMR spectra for the calcined xCe-MCM-41_US materials are shown in Figure 3d. All samples present a broad signal ranging from −90 to −120 ppm chemical shift values. To obtain individual information concerning the environment of the silicon nuclei in the structure, deconvolution of these signals was performed around the chemical shift values of −112, −101 and −92 ppm, for Q^4^, Q^3^ and Q^2^ species. A Gaussian/Lorentzian distribution ratio of 0.7 was chosen, to better fit the typical nuclear magnetic resonance spectra of solid samples. Table S1 summarizes the *Q^n^* distribution for all samples. To compare the *Q^n^* values among samples, the total silanol/siloxane ratio ((Si(OSi)_3_OH)+Si(OSi)_2_(OH)_2_)/(Si(OSi)_4_) and isolated silanol/siloxane ratio (Si(OSi)_3_OH)/(Si(OSi)_4_) were calculated. Bare silica MCM-41 shows the highest amount of both geminal and isolated groups, and their amount decreases when Ce is incorporated into the silica network. The isolated silanol/siloxane ratio is 0.76 for the unmodified mesoporous material and Ce incorporation results in a variable reduction ranging from 0.36 to 0.45. To a lesser extent, the amount of geminal silanols (Si(OSi)_2_(OH)_2_) also accounts for the total silanolic sites in the material. For unmodified silica, the recurrence of total silanol (*Q^3^+Q^2^*) is comparable to that of siloxane groups (*Q^4^*). The reduction of both ratios could be indicative of a successful isomorphic incorporation and provides further evidence of the substitution of Si for Ce as terminal atoms during oligomer polymerization and mesostructure formation. The presence of isomorphically bonded Ce heteroatoms could modify the silicon environment in the hexagonal framework by promoting the formation of Si-O-Ce, Ce-O-Ce and Ce-OH in the structure. These results agree with the spectroscopic and X-ray characterization already shown.

**Figure 4:**
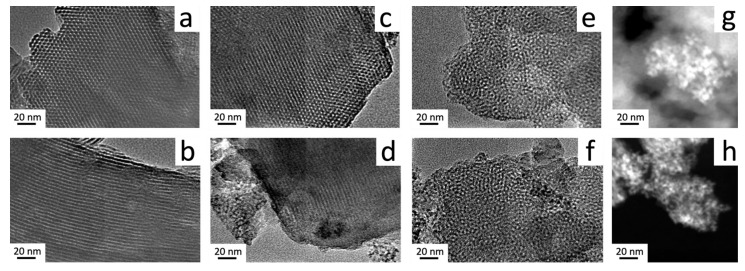
HR-TEM: (**a**) Cross sectional area of the hexagonal structure and (**b**) view along the channels for MCM-41; (**c**) Hexagonal structure and (**d**) interplanar distance for 0.02Ce-MCM-41_US; (**e**) 0.04Ce-MCM-41_US and (**f**) 0.06Ce-MCM-41_US; (**g**) High-Angle Annular Dark-Field Imaging for CeO_2_ particles in 0.08Ce-MCM-41_US and (**h**) 0.06Ce-MCM-41_US. (Bar length = 20nm for all micrographs).

Transmission electron micrographs shown in Figure 4 provide supplementary evidence of the hexagonal structure, characterizing the Ce modified mesoporous silica. For unmodified mesoporous sieves, two different observation angles are featured. The cross-sectional area offers a view of the hexagonal arrangement (Figure 4a) and a second view along the channels of the mesoporous material (Figure 4b) confirms the interplanar d_100_ distance calculated by SAXS. As the material loses the degree of ordering, the interplanar distance and the pore size enlarge because of the amount of incorporated cerium in the sample. For all mesoporous solids, a honeycomb-like structure is observed. High-Angle Annular Dark-Field Imaging of the sample with the highest cerium contents (Figure 4g,h) features medium size CeO_2_ domains condensed outside the silicon framework, which are not found in samples with lower Ce/Si ratios. In close agreement with the results obtained by X-ray diffraction, Bragg scattering from these ceria particles is associated with the presence of cubic tetracoordinated Ce species.

### 3.2. Ce-Modified Hexagonal Silica as Catalytic Supports for Nickel

**Figure 5:**
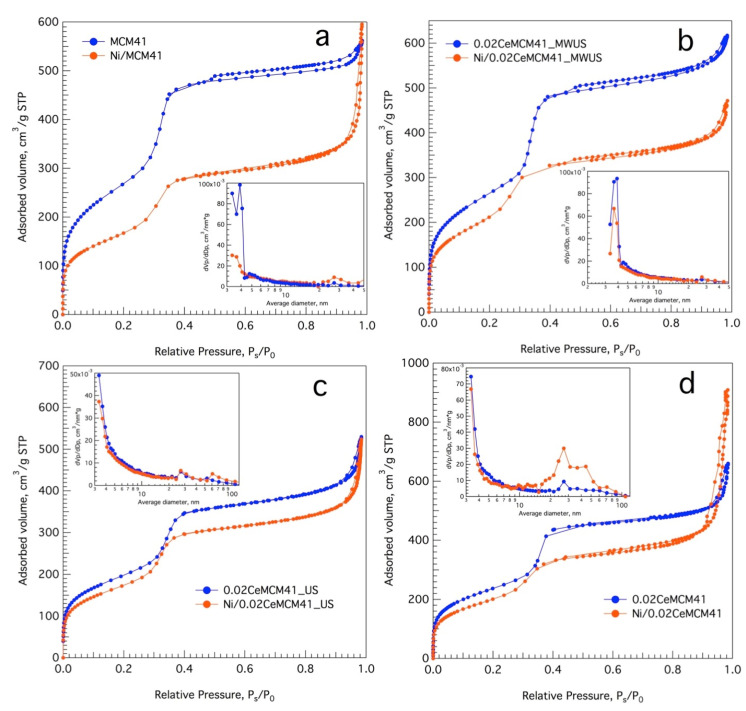
N_2_ isotherms for nickel catalysts (10% wt) over Ce-promoted mesoporous silica sieves: (**a**) MCM-41; (**b**) 0.02Ce-MCM-41_MWUS; (**c**) 0.02Ce-MCM-41_US and (**d**) 0.02Ce-MCM-41 (blue curves: bare supports; orange curves: Ni catalysts; insets: BJH pore size distribution graphs for both samples).

The cerium promoted mesoporous sieves were used as support for a Ni active phase (10% metal load). Nitrogen adsorption-desorption isotherms were recorded for calcined powders before and after Ni loading. Figure 5 shows the N_2_ isotherms and pore size distribution graphs for selected samples. According to the classification made by IUPAC [38], all prepared materials exhibit a type IV isotherm, characteristic of mesoporous materials ranging from 2 to 50 nm in pore size. For all catalytic supports, four well-defined regions in the isotherm can be identified: (a) A large nitrogen uptake at very low relative pressure values (P_s_/P_0_ up to 0.02), that can be associated to micropore filling and monolayer formation; (b) An inflexion point and slope change, followed by a more linear region in the isotherm curve is indicative of unrestricted multilayer adsorption; (c) A second inflexion point at relative pressure values close to 0.30 with a significant amount of adsorbed nitrogen is due to capillary condensation within the mesopores—this value is closely related to the pore diameter; (d) a final saturation plateau once the pores are filled is associated to adsorbed nitrogen on the outer surface of the material and in between particles. After Ni loading, the isotherm form is kept, and no significant area reductions occur, suggesting that nickel is well distributed and does not produce pore blocking. For Ni/MCM-41 and Ni/0.02Ce-MCM-41 a final N_2_ uptake at P_s_/P_0_ values close to 1.0 could be indicative of nitrogen adsorption on the surface of sintered particles that does not contribute significantly to the surface area calculations. Figure S2a–d (Electronic Supplementary Information file) shows the isotherm and pore size distribution for cerium-modified mesoporous silicas prepared at higher Ce/Si molar ratios.

The Barrett–Joyner–Halenda pore size distribution graphs (inset Figure 5a–d and Figure S2a–d), show a monomodal and sharp graph distribution for mesoporous solids prepared at low Ce/Si molar ratios while a second broad pore size within the mesopore range (close to 40 nm) can be observed for samples with the highest cerium content (0.06 and 0.08). Since pore size largely depends on the length of the surfactant alkyl chain and the possible presence of micelle swelling agents [28], this bimodal BJH pore graph could only be attributed to interparticle sintering due to calcination.

A summary of the textural properties for all materials is reported in Table 2. The calculations for the surface area were performed with the Brunauer–Emmett–Teller method. Unmodified mesoporous MCM-41 features the highest surface area (980 m^2^g^−1^), the narrowest pore size (3.49 nm, assuming a system of cylindrical pores with open endings), the highest monolayer capacity (225 m^2^g^−1^) and pore volume (0.86 cm^3^g^−1^). For the cerium-promoted mesoporous silicas, the largest surface area was obtained when microwave irradiation was used for thermal activation during hydrothermal synthesis, which can be attributed to a more uniform temperature profile produced by MW bulk heating. The absence of temperature inhomogeneities and hot spots (Figure S1, ESI file), obtained using the innovative coaxial MW applicator, could trigger a uniform particle growth during silica polymerization under solvothermal treatment. The sample with the highest Ce/Si molar ratio has a surface area of 546 m^2^g^−1^, while the pore size expands to a final value of 4.39 nm and the monolayer adsorptive capacity is reduced to 125 m^2^g^−1^. Nonetheless, all synthesized materials exhibit areas well above the values reported elsewhere for ceria and ceria/silica as catalytic supports [39,40], or those obtained with sol-gel methodologies. As the pore size enlarges, a reduction in the capillary forces in the material could occur, and the total pore volume decreases to 0.60 cm^3^g^−1^. In any case, the pore shape is conserved regardless of the pore broadening, as the BJH graphs still feature a sharp main peak. This effect can also be observed in a reduced nitrogen uptake in the capillary condensation region for samples with higher Ce/Si molar ratios (Figure S2a–d, relative pressure P_s_/P_0_ ~ 0.24 and inset BJH graphs).

**Figure 6:**
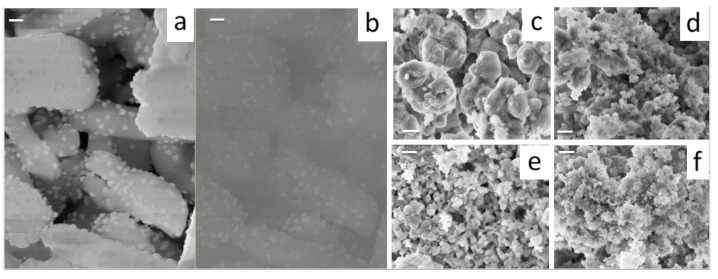
SEM Micrographs for 10% Ni/MCM-41: (**a**) Obtained using the In-lens detector. (**b**) Z-contrast image acquired using backscattered electrons. Particle morphology for: (**c**) Ni/0.02Ce-MCM-41_US, (**d**) Ni/0.04Ce-MCM-41_US, (**e**) Ni/0.06Ce-MCM-41_US and (**f**) Ni/0.08Ce-MCM-41_US catalysts. (Bar length = 200 nm, magnification 100 kX for all micrographs).

To characterize the nickel deposition and particle size for the mesoporous silica supported Ni catalysts, Scanning Electron Micrographs were acquired using dual detection for secondary and backscattered electrons to yield Z-contrast images of the Ni active phase on the catalyst surface (Figure 6a,b). The wet impregnation method enabled the formation of highly dispersed Ni clusters using the cerium promoted sieves as support. Although the dispersion of Ni catalyst on pure siliceous MCM-41 was recently reported by co-condensation [41] and co-impregnation [42] methods, the effect of cerium isomorphic incorporation on the particle size of the Ni clusters is still poorly understood. Regarding particle morphology, elongated club-like particles can be observed for the parent material. As the amount of cerium increases in the sample, a silica polymerization disruptive effect can be observed. Ce isomorphic incorporation could hinder silica self-assembly of the micellar aggregates, limiting particle growth, which produced a different morphology for Ce-promoted silicas. For intermediate Ce content, shorter round-like particles can be observed, whereas samples prepared at the highest Ce/Si molar ratio resulted in flake-like morphologies. The presence of the heteroatom seems to limit nickel particle growth and deposition, since Ni particles are easily observed in pure MCM-41 (Figure 6a,b) that were not distinguished in other materials.

### 3.3. Catalytic Evaluation of Ni/Ce-Promoted Silica in the Ethanol Steam Reforming Reaction

The performance of xCe-MCM-41 supported nickel catalysts was evaluated in the ethanol steam reforming reaction (ESR). The overall reaction is:

C_2_H_5_OH + 3H_2_O → 6H_2_ + 2CO_2_ (1)

**Figure 7:**
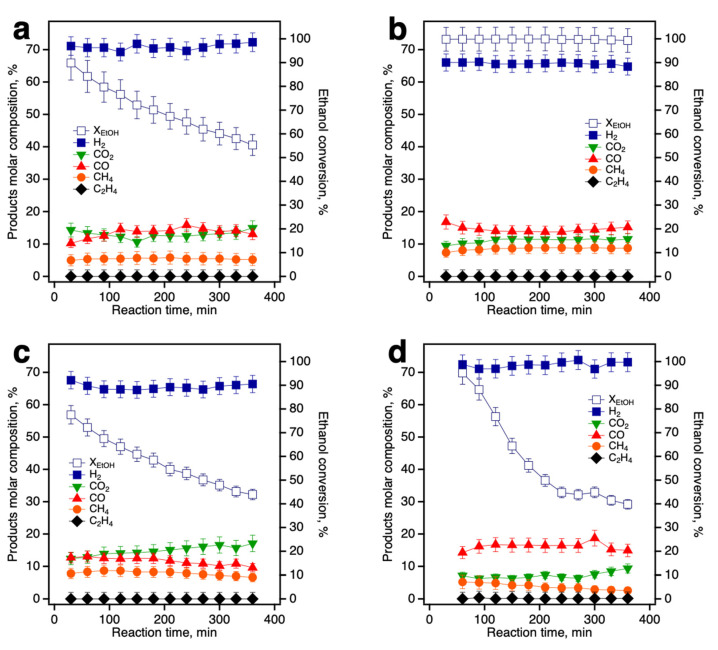
Ethanol conversion (right axis, open markers) and product distribution (left axis, full markers) for Ni/xCe-MCM-41 catalyst in the ethanol steam reforming reaction: (**a**) Ni/MCM-41 at 773 K; (**b**) Ni/0.02Ce-MCM-41_MWUS at 773 K; (**c**) Ni/0.02Ce-MCM-41_MWUS at 723 K; (**d**) Ni/0.02Ce-MCM-41 (Conventional synthesis, no US) at 773 K.

For the Ni/xCe-MCM-41 catalyst series, Figure 7 reports ethanol conversion and product distribution vs. reaction time. The molar composition for the individual species (y_i_) was calculated in terms of all formed products (Σ_j_F_j_), as y_i_ = F_i_/Σ_j_F_j_. The conversion of ethanol was obtained using the value of the fluxes at the entrance and exit of the reactor: X_EtOH_(%) = [(F_EtOH_,_in_ – F_EtOH_,_out_)/F_EtOH_,_in_] × 100. According to a proposed reaction mechanism [43], some of the side reactions that can occur during the ESR are ethanol cracking to C1 products (Equation (2)), followed by steam reforming of CH_4_ (Equation (3)):

C_2_H_5_OH + H_2_O → CH_4_ + CO + H_2_ (2)

CH_4_ + 2H_2_O → 4H_2_ + CO_2_ (3)

Catalyst deactivation by carbon poisoning is mainly produced by CH_4_ decomposition (Equation (4)), Boudouard reaction (Equation (5)) and ethanol dehydration to ethylene (Equation (6)), followed by surface polymerization and C deposition.

CH_4_ → 2H_2_ + C (4)

2CO → CO_2_ + C (5)

C_2_H_5_OH → C_2_H_4_ + H_2_O (6)

When unmodified silica MCM-41 was used as catalytic support, the Ni/MCM-41 catalyst deactivated swiftly after 6h on stream and ethanol conversion decreased to a final value of 52%. Molar fraction for all products remained constant and was calculated to be: 70% for H_2_, 13% for CO_2_, 12% for CO, 4% for CH_4_ and traces of C_2_H_4_ (Figure 7a). This catalyst presents the largest Ni particle size and is prone to deactivation by coke deposition and sintering as reported by Parlett et al. [44]. On the other hand, when Ce-promoted silica was used as support (0.02Ce-MCM-41_MWUS, Figure 6b), complete hydrogen conversion was achieved at 773 K, while product distribution was 65% H_2_, 11.4% for CO_2_, 15% for CO, 8.6% for CH_4_, with traces of C_2_H_4_. A lower methane molar fraction for bare silica as support could be indicative that methane decomposition is favored on the catalyst surface, which slightly increases the H_2_ yield. For all Ce-containing materials, full ethanol conversion was achieved at 773 K (Electronic Supplementary Information, Table S2), and the main product distribution and yield did not change with Ce content. Methane selectivity ranged from 8.5 to 10%, and no traces of acetaldehyde or C2 products were detected. Similar selectivity in all these materials is indicative of a common reforming mechanism which is independent of nickel particle size [44]. The stability and ethanol conversion of the Ce-promoted mesoporous silica can be attributed to oxygen storage capacity and the redox conversion of Ce^4+^ ↔ Ce^3+^ in the catalyst surface. The reduced Ce^3+^ sites produce H_2_O dissociation to form OH^-^ surface groups, which catalyze the reforming of C1 species (Equation (3)). This reaction pathway was reported for a Ni catalyst supported on cubic CeO_2_ [45].

To analyze the ethanol conversion and hydrogen selectivity at a lower temperature level, the ESR was carried out on 0.02Ce-MCM-41_MWUS at 723 K (Figure 7c). While a similar product distribution was observed, the Ni catalyst was less active at this temperature as ethanol conversion dropped to 44% after 6h on stream. H_2_ selectivity was above 65% and the catalyst yielded lower CO and CH_4_ in the products stream (compared to the same catalyst at 773 K), suggesting that after ethanol cracking, C1 products are carbonized via methane decomposition and Boudouard reactions (Equations (4) and (5)). For comparative purposes, a sample prepared with conventional hydrothermal synthesis without US micromixing for Ce dispersion was also used as catalytic support. For this material, a swift catalyst deactivation was also observed. In Figure 1a it was observed that this catalyst did not retain the silica framework, so its behavior is equivalent to an amorphous Ni/CeO**_2_**-SiO**_2_**. After achieving full ethanol conversion during the first hour on stream, the final value was below 40%. Product distribution was found to be: 73% for H_2_, 15% for CO, 9% for CO_2_ and 2% for CH_4_. A higher carbon monoxide yield, compared to the US-synthesized supports, could be indicative of a poor distribution of the Ce species and, therefore, less available surface sites that do not catalyze complete oxidation from CO to CO_2_. Carbon deposition for this sample could be associated with methane decomposition, since CH_4_ molar concentration was the lowest for the catalyst series. A further screening on the Ce/Si ratios for the catalytic support and Ni metal loading is beyond the scope of this study since the properties of the parent material MCM-41 are no longer preserved.

**Figure 8:**
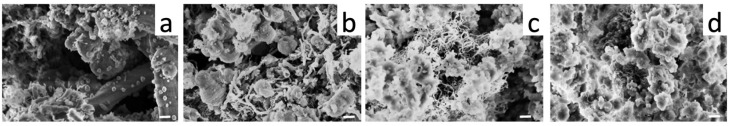
Nanocarbon filaments formation over: (**a**) Ni/MCM-41, (**b**) Ni/0.02Ce-MCM-41_US, (**c**) Ni/0.04Ce-MCM-41_US and (**d**) Ni/0.06Ce-MCM-41_US after 6 h on reaction stream (Bar length = 200 nm, magnification 100 kX).

Figure 8 shows the graphitic carbon formation on Ni/xCe-MCM-41_US catalysts. For all Ce-promoted silicas, only traces of ethylene were observed (below 0.1%), which suggest that after ethanol dehydration (Equation (6)), any formed ethylene could quickly polymerize, resulting in carbon formation and deposition. Nevertheless, the Ce modified mesoporous silica provides a suitable matrix for the immobilization of the Ni species since no metallic sintering was observed. The distribution of the Ce species in the hexagonally ordered catalytic sieve resulted in formation of carbon nanofibers rather than aggregated carbon. As transition metal catalysts provide a suitable substrate for the growth of ordered carbon nanorods and nanofilaments [46], the hexagonal structure, adequate Ce dispersion and surface features of Ce-MCM-41 resulted in a controlled growth of carbon nanofibers that did not produce catalyst deactivation by carbon poisoning. Cerium incorporation favors the formation of small carbon filaments that do not block active sites (Figure 8), even at the lowest cerium concentration used in the present research.

## 4. Conclusions

Cerium isomorphic incorporation on a mesoporous silica structure was achieved by US-assisted micromixing during micellar formation. This sonochemical approach enhanced Ce dispersion and preserved the hexagonal ordering at high Ce/Si molar ratios (Ce/Si = 0.08). Further, an innovative coaxial applicator was used for in-situ MW irradiation as an alternative to conventional heating for solvothermal synthesis. The combined use of US and MW irradiation represents a green synthesis approach, as the overall reaction time was reduced, and the precursor reagents were employed efficiently. These cerium-modified materials were used as a nickel catalyst support for the ethanol steam reforming reaction to produce hydrogen. The sonochemical approach coupled with dielectric heating resulted in a better catalytic support for the ESR reaction compared to the same Ni catalyst over mesoporous silica synthesized by magnetic stirring and conventional heating. The new catalyst featured complete ethanol conversion, high H_2_ selectivity (65%) and no catalyst deactivation occurred. Catalyst stability (6 h on stream) during the ESR reaction at 773 K was closely related to the adequate cerium distribution along the silica matrix. The presence of the heteroatom in the mesoporous sieve modified the morphology and chemical behavior of the catalyst, enabling an ordered deposition of carbon nanofilaments that do not induce catalyst deactivation or catalyst poisoning.

The US and MW-assisted green synthesis reported here can be easily implemented for other types of solvothermal synthesis routes that make use of amphiphilic molecules as structure-directing agents.

## Figures and Tables

**Table 1:** Lattice cell parameters for Ce-modified mesoporous silica sieves.

Sample Name	Ce/Si Ratio	d_100_, nm ^[a]^	a_0_, nm ^[b]^
MCM-41	0	4.42	5.10
0.02Ce-MCM-41_MWUS ^[c]^	0.02	4.46	5.15
0.02Ce-MCM-41 ^[d]^	0.02	4.78	5.52
0.02Ce-MCM-41_US ^[e]^	0.02	4.63	5.35
0.04Ce-MCM-41_US ^[e]^	0.04	4.88	5.63
0.06Ce-MCM-41_US ^[e]^	0.06	4.98	5.75
0.08Ce-MCM-41_US ^[e]^	0.08	5.04	5.82

[a] Interplanar spacing. [b] Lattice cell constant, a_0_ = 2d_100_/√3. [c] Synthesized using US for micromixing and MW for thermal activation. [d] Conventional ageing, using magnetic stirring. [e] Synthesized using US during colloidal ageing.

**Table 2:** Textural properties for Ce- modified mesoporous silica.

Sample Name	S_BET_ ^[a]^, m^2^g^−1^	V_pore_ ^[b]^, cm^3^g^−1^	V_monolayer_ ^[c]^, cm^3^g^−1^	D_pore_ ^[d]^, nm
MCM-41	980	0.86	225	3.49
0.02Ce-MCM-41_MWUS	967	0.94	222	3.88
0.02Ce-MCM-41	865	0.96	199	4.45
0.02Ce-MCM-41_US	806	0.77	185	3.84
0.04Ce-MCM-41_US	711	0.68	163	3.84
0.06Ce-MCM-41_US	629	0.65	145	4.14
0.08Ce-MCM-41_US	546	0.60	125	4.39

[a] S_BET_, surface area calculated with the Brunauer–Emmett–Teller model. [b] V_pore_, total pore volume, estimated at the relative pressure Ps/Po = 0.9814. [c] V_monolayer_ monolayer volume expressed at STP. [d] D_pore_, pore diameter calculated as D = 4 × V_pore_/S_BET_.

## Data Availability

Datasets analyzed or generated during the study are available upon request to the corresponding authors.

## Supplementary Materials

The following supporting information can be downloaded at: https://www.mdpi.com/article/10.3390/nano13060997/s1, Figure S1: Temperature profile for the MW-assisted hydrothermal synthesis of 0.02Ce-mesoporous silica. Figure S2: Nitrogen adsorption and desorption isotherms for (a) 0.02Ce-MCM-41_US, (b) 0.04Ce-MCM-41_US, (c) 0.06Ce-MCM-41_US and (d) 0.08Ce-MCM-41_US materials after calcination. Table S1: Q^n^ distributions for the Silicon species in xCe-MCM-41_US calcined materials. Table S2. Ethanol conversion (X_EtOH_) and product yield after 1 and 6h for the Ni/xCe-MCM-41 catalysts series in the ethanol steam reforming reaction (all reactions were performed at T = 773 K).
