# Supplementary material for: Ultrasound and Microwave-Assisted Synthesis of Hexagonally Ordered Ce-Promoted Mesoporous Silica as Ni Supports for Ethanol Steam Reforming
Source: Nanomaterials (Basel). 2023 Mar 9;13(6):997. doi: 10.3390/nano13060997 (PMC10053107; doi:10.3390/nano13060997)
Supplement: Supplementary file 1 [file nanomaterials-13-00997-s001.zip › nanomaterials-2242208-supplementary.pdf]

Electronic Supplementary Information

Ultrasound and microwave-assisted synthesis of hexagonally ordered Ce-promoted mesoporous silica as Ni supports for ethanol steam reforming

Jorge Tovar-Rodriguez[1], Emiliano Fratini[1,*], Piero Baglioni[1], Carlo Ferrari[2], José Antonio de los Reyes-Heredia[3], Yonatan Ramírez-Hernández[4] and Ignacio René Galindo-Esquivel[4,*]

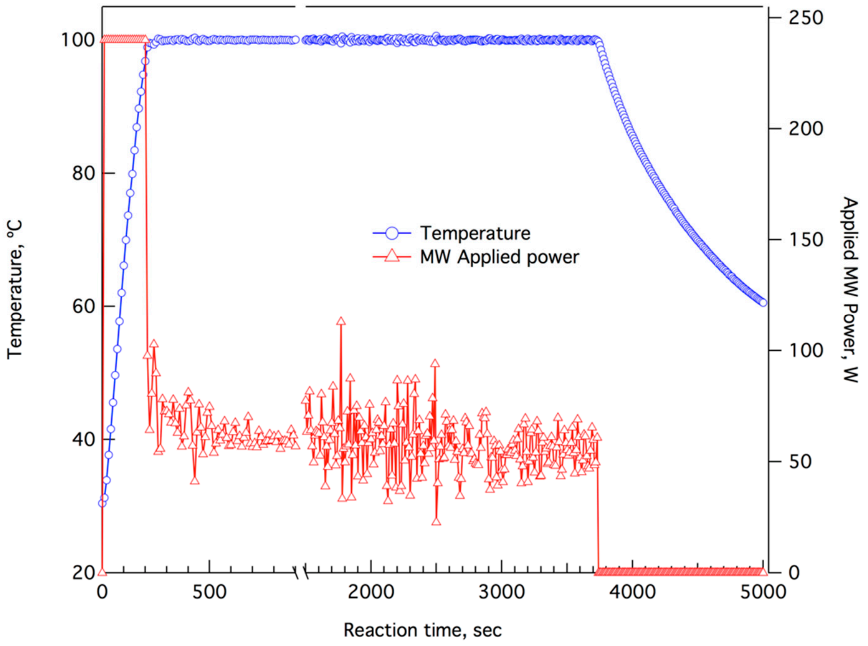

Figure S1. Temperature profile for the MW-assisted hydrothermal synthesis of 0.02Ce-mesoporous silica.

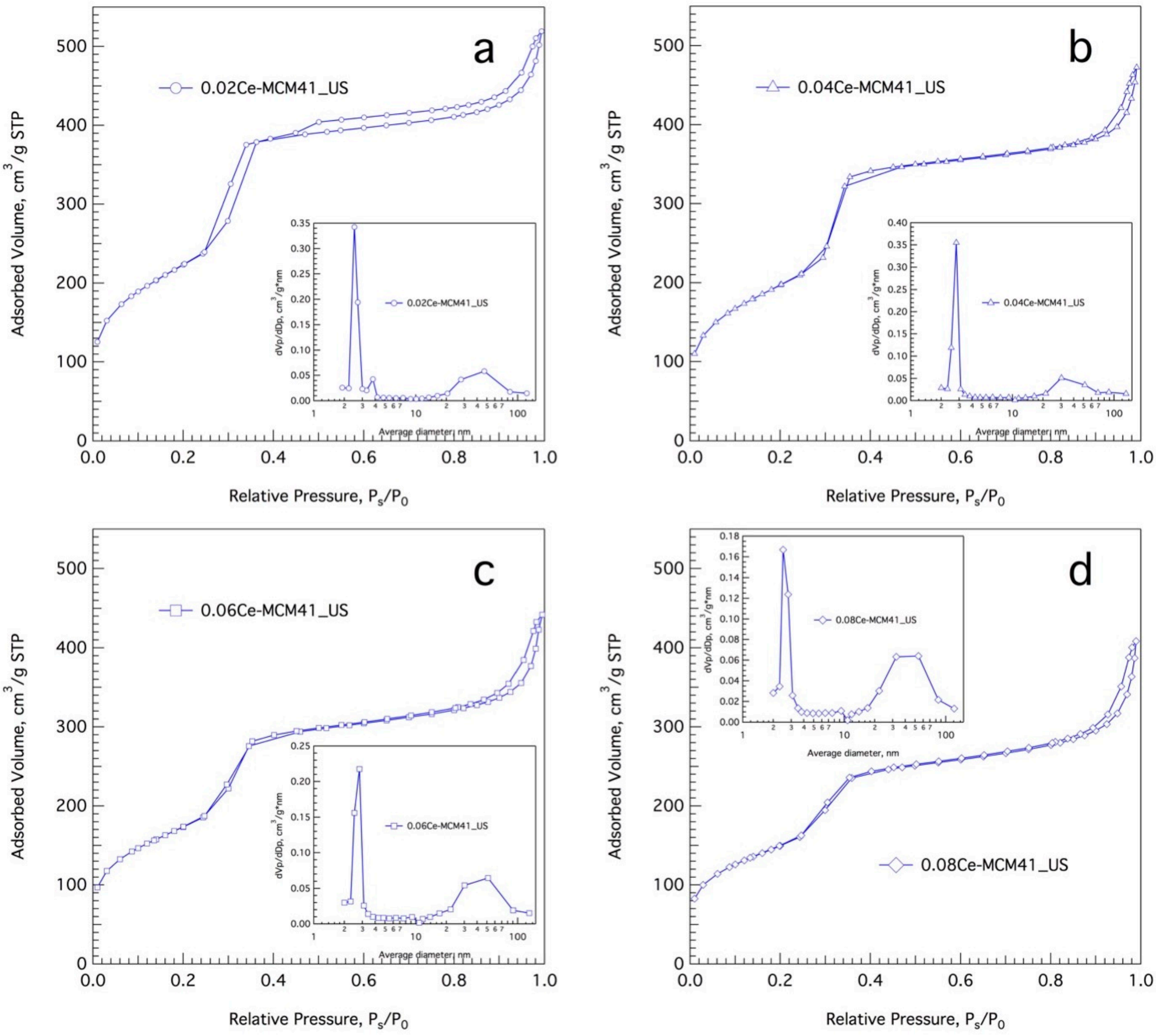

Figure S2. Nitrogen adsorption and desorption isotherms for a) 0.02Ce-MCM-41_US, b) 0.04Ce-MCM-41_US, c) 0.06Ce-MCM-41_US and d) 0.08Ce-MCM-41_US materials after calcination. Inset graphs: BJH pore size distribution for the same materials.

Table S1. Q^n distributions for the Silicon species in xCe-MCM-41_US calcined materials.

Sample	Q^4	Q^3	Q^2	Q^3/Q^4	$(Q^2+Q^3)/Q^4$
MCM-41	50.4	38.4	11.2	0.76	0.99
0.02Ce-MCM-41_US	62.9	22.6	14.5	0.36	0.59
0.04Ce-MCM-41_US	59.2	23.1	17.7	0.39	0.69
0.06Ce-MCM-41_US	59.6	26.6	13.8	0.45	0.68
0.08Ce-MCM-41_US	57.9	21.0	20.9	0.36	0.72

Table S2. Ethanol conversion (X_{EtOH}) and product yield after 1 and 6h for the Ni/xCe-MCM-41 catalysts series in the ethanol steam reforming reaction (all reactions were performed at T = 773 K).

Catalyst	X_{EtOH} at 1h	Product yield after 1 h				X_{EtOH} at 6h	Product yield after 6 h			
		H_2	CH_4	CO	CO_2		H_2	CH_4	CO	CO_2
Ni/0.02Ce-MCM-41_US	100	3.02	0.74	0.33	0.94	100	3.48	0.48	0.27	1.25
Ni/0.04Ce-MCM-41_US	100	3.11	0.58	0.35	1.08	100	3.38	0.55	0.33	1.12
Ni/0.06Ce-MCM-41_US	100	3.13	0.59	0.39	0.98	100	3.49	0.56	0.33	1.11
Ni/0.08Ce-MCM-41_US	100	3.38	0.51	0.30	1.10	100	3.14	0.47	0.34	1.19
